# Event-driven contrastive divergence: neural sampling foundations

**DOI:** 10.3389/fnins.2015.00104

**Published:** 2015-03-31

**Authors:** Emre Neftci, Srinjoy Das, Bruno Pedroni, Kenneth Kreutz-Delgado, Gert Cauwenberghs

**Affiliations:** ^1^Integrated Systems Neuroengineering Laboratory, Institute for Neural Computation, University of California, San DiegoLa Jolla, CA, USA; ^2^Electrical and Computer Engineering Department, University of California, San DiegoLa Jolla, CA, USA; ^3^Department of Bioengineering, University of California, San DiegoLa Jolla, CA, USA

**Keywords:** neural sampling, spiking neurons, synaptic plasticity, Markov chain Monte Carlo, probabilistic inference

In a recent Frontiers in Neuroscience paper (Neftci et al., 2014) we contributed an on-line learning rule, driven by spike-events in an Integrate and Fire (IF) neural network, that emulates the learning performance of Contrastive Divergence (CD) in an equivalent Restricted Boltzmann Machine (RBM) amenable to real-time implementation in spike-based neuromorphic systems. The event-driven CD framework assumes the foundations of *neural sampling* (Buesing et al., 2011; Maass, 2014) in mapping spike rates of a deterministic IF network onto probabilities of a corresponding stochastic neural network. In Neftci et al. (2014), we used a particular form of neural sampling previously analyzed in Petrovici et al. (2013)^1^, although this connection was not made sufficiently clear in the published article. The purpose of this letter is to clarify this connection, and to raise the reader's awareness to the existence of various forms of neural sampling. We highlight the differences as well as strong connections across these various forms, and suggest applications of event-driven CD in a more general setting enabled by the broader interpretations of neural sampling.

In the Bayesian view on neural information processing, the cognitive function of the brain arises from its ability to encode and combine probabilities describing its interactions with an uncertain world (Doya et al., 2007). A recent neural sampling hypothesis has shed light on how probabilities may be encoded in neural circuits (Fiser et al., 2010; Berkes et al., 2011). In the neural sampling hypothesis, spikes are viewed as samples of a target probability distribution. From a modeling perspective, a key advantage of this view is that learning in spiking neural networks becomes more tractable than the alternative one, in which neurons encode probabilities, because one can borrow from well-established algorithms in machine learning (Fiser et al., 2010) (see Nessler et al., 2013 for a concrete example).

Merolla et al. (2010) demonstrated a Boltzmann machine using IF neurons. In this model, spiking neurons integrate Poisson-distributed spikes during a fixed time window set by a global rhythmic oscillation. A first-passage time analysis shows that the probability that a neuron spikes in the given time window follows a logistic sigmoid function consistent with a Boltzmann distribution. The particular form of rhythmic oscillation ensures that, even when neurons are recurrently coupled, the network produces a sample of a Boltzmann distribution for each oscillation cycle. Merolla et al. (2010) also suggest an alternative, more biologically plausible forms of learning induced by rhythmic oscillations that resemble the role of theta oscillations across large neuronal ensembles. Our event-driven CD rule is compatible with Merolla et al.'s sampler because it would simply result in updating weights at every cycle of the rhythmic oscillation.

Shortly after, Buesing et al. (2011) proved that abstract neuron models consistent with the behavior of biological spiking neurons (Jolivet et al., 2006) can perform Markov Chain Monte Carlo (MCMC) sampling of a Boltzmann distribution. Their sampler does not require global oscillations, although these could enable the sampling from multiple distributions within the same network (Habenschuss et al., 2013). To demonstrate the performance of the sampler, a Boltzmann machine was trained off-line using CD. Learning in this model was further extended to on-line updates in a precursor of event-driven CD (Pedroni et al., 2013).

An open question was whether neuron models that describe the biological processes of nerve cells endowed with deterministic action potential generation mechanisms can support stochastic sampling as described with the more abstract spiking forms in Buesing et al. (2011). An answer to this question is relevant for understanding how neural sampling can be instantiated in biological neurons, but also for implementing neural samplers on low-power neuromorphic implementations of spiking neurons (Indiveri et al., 2011). The stochastic nature of neural sampling suggests studying the behavior of neurons under noisy inputs. The diffusion model commonly referred to as the Ornstein-Uhlenbeck process (Van Kampen, 1992) has been the basis of a standard continuous-time stochastic neuron model since the first rigorous analysis of its behavior in Capocelli and Ricciardi (1971). Petrovici et al. (2013) discuss these issues and provide a rigorous link between deterministic neuron models (leaky integrate-and-fire with conductance-based synapses) and stochastic network-level dynamics, as can be observed *in vivo*. In particular, they identify how the high-conductance state caused by Poissonian background bombardment can provide the fast membrane reaction time required for precise sampling. They provide analytical derivations of the activation function at the single-cell level as well as for the synaptic interaction and investigate the convergence behavior of the sampled distribution at the network level.

O'Connor et al. (2013) employ the Siegert approximation of IF neurons to compute CD updates. The Siegert or diffusion approximation expresses the firing rate of an IF neuron, as a function of input firing rates, under the assumption that all inputs are independent and Poisson distributed. After learning, the parameters of the learned Boltzmann machine are transferred to the equivalent network of IF neurons. Although the off-line CD learning in O'Connor et al. (2013) operated using firing rates rather than spikes, in its basic form, it is functionally equivalent and compatible with event-driven CD under the condition that spike times are uncorrelated.

Our work implements a biologically-inspired algorithm for the purposes of training Boltzmann machines (Neftci et al., 2014). We assumed a neuronal model consistent with biology and realizable in a neuromorphic implementation. Petrovici et al. (2013) provided a deeper physical and mathematical interpretation of neural sampling. Similarly to their approach, we considered the standard leaky IF neuron stimulated by non-capacitively summed pre-synaptic inputs obeying Poisson statistics.

The performance of event-driven CD on the MNIST hand-written digit recognition task was robust to spike probabilities that deviate slightly from the Boltzmann distribution, even though such distributions violate the assumptions of CD formulated for training RBMs. This suggests that event-driven CD provides a general learning framework for biologically-inspired spiking RBMs and is consistent with wide range of neural samplers.

## Conflict of interest statement

The authors declare that the research was conducted in the absence of any commercial or financial relationships that could be construed as a potential conflict of interest.

## Footnotes

^1^A functionally equivalent formulation can be found in an earlier version posted on arXiv (Neftci et al., 2013)
